# The need to implement non-industry COVID-19 clinical trials in non-high-income countries

**DOI:** 10.7189/jogh.10.010351

**Published:** 2020-06

**Authors:** Rafael Dal-Ré, Antonio J Carcas, Nadarajah Sreeharan

**Affiliations:** 1 Epidemiology Unit, Health Research Institute-Fundación Jiménez Díaz University Hospital, Universidad Autónoma de Madrid, Madrid, Spain; 2 Clinical Pharmacology Department, IdiPAZ, La Paz University Hospital School of Medicine, Universidad Autónoma de Madrid, Madrid, Spain; 3 Department of Medicine, University of Jaffna, Jaffna, Sri Lanka

The COVID-19 pandemic is currently being managed globally using public health measures to contain and mitigate its impact. The COVID-19 spares no national boundaries and is spreading rapidly in both high-income and non-high-income countries [1]. The lack of approved therapies and the absence of a vaccine for COVID-19 have led to the repositioning of some existing medications with well-defined benefit-risk profiles in other indications. It is important to note that none of these medications have sufficient evidence on benefit-risk in COVID-19 patients, and should therefore be considered as experimental therapies, with an urgent need to collect data that will facilitate or prevent their use in COVID-19 patients – something that is already happening in many countries [2].

We searched four databases (ANZCTR, ClinicalTrials.gov, EU-CTR, ISRCTN) looking for randomized controlled trials (RCTs) aiming to assess the comparative efficacy of different treatment drug regimens for COVID-19 hospitalized patients, sponsored by non-industry institutions/organizations based in high-income countries, that were registered on April 3, 2020. Two searches were conducted in each database using the terms ’coronavirus’ and ‘COVID-19’ as descriptors. In the two searches on ClinicalTrials.gov, we also used the following descriptors: Study type: ‘Interventional studies (clinical trials)’; Status: recruitments: ‘Not yet recruiting’ and ‘recruiting’; Funder type: ‘NIH’, ‘Other US Federal Agency’ and ‘All others (individuals, universities, organizations)’. **Table 1** shows the main characteristics of the eleven largest of these RCTs. The experimental medicines in all these trials are to be administered on top of the available standard of care.

**Table 1:** Largest non-industry randomized controlled trials sponsored by organizations/institutions based on high-income-countries assessing experimental medicines (vs standard of care, with or without placebo) to treat COVID-19 hospitalized patients, that were recruiting or about to start enrollment; trials first registered on ANZCTR, ClinicalTrials.gov, EudraCT (accessed through EU-CTR) or ISRCTN, as of April 3, 2020

Name/Trial ID	Country (ies)/Sponsor	Treatments†	N	Design/status	Primary outcome measures*
ACT COVID 19 / NCT04324463	France/Public assistance, Paris Hospitals	Azithromycin + chloroquine vs SOC	1500	2-arm; parallel; open-label/Not yet recruiting	Inpatients: invasive mechanical ventilation or mortality.
					Out-patients: hospital admission or death; up to 6 weeks post-randomization
ACTT / NCT04280705, 2020-01052-18	Denmark, Germany, Japan, Korea, Singapore, Spain, UK, USA/NIAID	Remdesivir vs placebo	572	2-arm; adaptive; parallel; double-blind; placebo-controlled/Recruiting	Percentage of subjects reporting each severity rating on an 8-point ordinal scale (from 1.death to 8.not hospitalized, no limitations on activities); at day 15
COLCOVID / NCT04328480	Argentina/ECLA	Colchicine ( ± lopinavir/ritonavir) vs SOC	2500	2-arm; parallel; open-label/Not yet recruiting	All-cause mortality, at day 30
CORIMUNO-19/2020-001246-18	France/Public assistance, Paris Hospitals	Sarilumab iv vs tocilizumab iv vs tocilizumab sc vs SOC	1000	4-arm; parallel; open-label/Recruiting	Non-ICU patients: Survival without ventilator, at day 14; and WHO progression scale < or = 5, at day 4
					ICU patients: Co-primary endpoints: 1. cumulative incidence of tracheal extubation, at day14; death, and 2. WHO progression scale >7, at day 4
COVID MED / NCT04328012	USA/Bassett Healthcare	Lopinavir/ritonavir vs hydrochloro-quine vs losartan vs placebo	4000	4-arm; parallel; double-blind/Not yet recruiting	NIAID COVID-19 ordinal severity scale, at day 60
DISCOVERY / 2020-000936-23, NCT04315948	Belgium, France, Germany, Luxembourg, the Netherlands, Spain, Sweden, UK/INSERM	Remdesivir vs Lopinavir/ritonavir ± IFNβ vs hydroxychloroquine vs SOC	3100	5-arm‡; adaptive; parallel; open-label/Recruiting	Clinical status on a 7-point ordinal scale (from 1. not hospitalized, no limitations of activities, to 7. death); at day 15
ENACOVID / 2020-001301-23, NCT04325633	France/Public assistance, Paris Hospitals	Naproxen vs SOC	584	2-arm; parallel; open-label/Not yet recruiting	All-cause mortality, at day 30
HYCOVID / 2020-001271-33, NCT04325893	France/Angers University Hospital	Hydroxychloroquine vs placebo	1500	2-arm; parallel; double-blind/Not yet recruiting	All-cause mortality or the use of intubation and invasive ventilation, at day 14
ORCHID / NCT04332991	USA/Massachusetts General Hospital	Hydroxychloroquine vs placebo	510	2-arm; parallel; double-blind/Not yet recruiting	Improvement on a 7-point ordinal scale (from 1. Death to 7. Not hospitalized, no limitations on activities), at day 15
RECOVERY / 2020-001113-21, ISRCTN50189673	UK/University of Oxford	Lopinavir/ritonavir vs dexamethasone vs IFNβ1a vs hydrochloroquine § vs SOC	5000	5-arm‖; adaptive; parallel; open-label/Recruiting	In-hospital mortality; at day 28 post-randomization
SOLIDARITY / 2020-001366-11, ISRCTN83971151	Argentina, Brazil, Canada, Germany, Indonesia, Iran, Norway¶, Peru, Qatar, South Africa, Spain, Switzerland, Thailand/WHO	Remdesivir vs Lopinavir/ritonavir ± IFNβ-1a vs Hydroxychloroquine or chloroquine vs SOC	Thou-sands**	5-arm‖; adaptive; parallel; open-label/Not yet recruiting	All-cause mortality (at discharge or death)

ECLA – Latin American clinical trials, Rosario, ICU – intensive care unit, IFNβ1a - interferon beta 1a, INSERM – French National Institute of Health and Medical Research, NIAID – US National Institute of Allergy and Infectious Diseases, SOC – standard of care, WHO – World Health Organization

*Mortality is a secondary outcome measure in ACTT, COVID MED, DISCOVERY and ORCHID. To be included in the table, the trial had to recruit, at least, 250 participants per arm. All RCTs that were described as triple- or quadruple-blind on ClinicalTrials.gov appear in the table as double-blind to be consistent with the EU-CTR terminology that only considers single- and double-blind trials.

†All experimental drug treatments and placebos are given on-top of standard of care.

‡It is not clear the number of arms in this trial since it has 4 on the EU-CTR (2020-000936-23; and 3300 participants) and 5 on ClinicalTrials.gov (NCT04315948; and 3100 participants).

§Hydroxychloroquine is mentioned in the EU-CTR and in the participant’s information sheet, although not in the study protocol [3].

‖If one or more drugs is not available or is contraindicated, random allocation will be adjusted between the remaining arms (2:1:1 or 2:1 ratio), as stated in the protocol [3].

¶It is not clear the number of arms that the trial will have in Norway. Thus, in the EU-CTR (2020-000982-18) the trial will assess only remdesivir vs hydroxychloroquine in 443 participants, but on ClinicalTrials.gov (NCT04321616), the trial will assess remdesivir vs hydroxychloroquine vs SOC in 700 participants.

**The ISRCTN registry (ISRCTN83971151) stated ‘several’ thousands, whereas the EU-CTR (2020-001366-11) stated 100 000 participants (which seems a too high number).

## THE STANDARD OF CARE ARM

At a time when emotions ride high and panic may prevail, it is important to strike the right balance in ensuring that, although public health and saving lives remain paramount, there is also the need to design RCTs in a manner that facilitates their conduct in both high-income and non-high-income countries, to ultimately ensure global value and relevance. We are concerned that patients in many countries will reject participation in trials with a control arm (receiving only the available standard of care, and placebo in masked trials), since the expectation will be to receive treatment with one of the experimental medicines. The distress that prolonged quarantine commonly produces [4] could be enhanced in a patient on hospitalization with a diagnosis of the COVID-19 infection, who is then invited to participate in a RCT with the chance of not receiving any of the experimental medications when these could be prescribed off-label or received through compassionate use or an expanded access program [2] by refusing to participate in the trial. The recent FDA approval of hydroxychloroquine sulfate and chloroquine phosphate, though limiting its usage for hospitalized COVID-19 patients when certain conditions are met [5], will further exacerbate this problem even outside the USA. Furthermore, heatlhcare professionals may be reluctant to get involved in trials in which their patients would receive only the standard of care, especially in sites where the number of severe hospitalized cases is high. All 49 trials found in our search, except eight, have a control arm (Appendix S1 in the **Online Supplementary Document**). The situation is made worse in RECOVERY by the anticipated random allocation ratio of 2:1:1:1, so participants will have a 40% chance of not receiving any experimental medication [3]. Although we recognize the need for a control arm to enhance the comparative efficacy results of these RCTs, it is also possible that the recruitment into these trials could be jeopardized with inadequate enrolment – albeit how the information regarding the current lack of evidence-based targeted treatments is provided to potential participants by investigators is the key factor to ensuring recruitment. In a pandemic, the critical need to care for current patients should not negatively impact on research that will benefit future patients [6].

**Figure:**
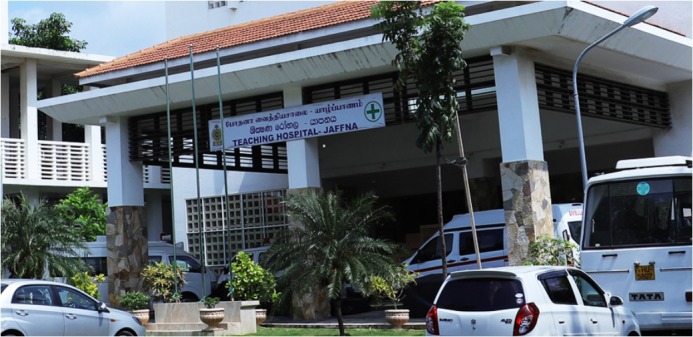
Photo: From the Jaffna Teaching Hospital, Jaffna; Sri Lanka (used with permission).

## MORTALITY AS PRIMARY OUTCOME MEASURE

Since the mortality rate in hospitalized patients with COVID-19 pneumonia is significant, especially in patients over 60 years of age [7], we believe that RCTs with mortality as the single primary outcome measure (as is the case in COLCOVID, ENACOVID, SOLIDARITY and RECOVERY) will have the most relevance as death is a) perceived as *the* key patient-centered endpoint; “saving lives” is the foremost message to encourage citizens adherence to the strict social distancing mandate; and b) a hard (most robust) outcome measure, which is the easiest to register especially in non-high-income, but also during pandemics in high-income countries. Assessment of other non-mortality-based outcomes will likely be a problematical exercise due to the extremely difficult and sustained situation of high stress and workload for all heatlhcare professionals [8]. When the emotional, mental and physical well-being of heatlhcare professionals is under extreme pressure, confounded by the continuous rotation of physicians and nurses caring for patients, seeking to register other primary and secondary outcome measures, may not only be feasible but also be unfair to the already exhausted heatlhcare professionals. Registration of non-mortality outcomes may not be an issue in hospitals with electronic health record systems, which should also be compatible with the central data management processes, something that could be an unsurmountable hurdle in international trials. We believe that even in heatlhcare systems that implement effective wellness activities to promote the resilience of heatlhcare professionals during the pandemic [9], the conduct of RCTs should not induce any additional adverse impact on their well-being.

Registering only deaths in international RCTs with an adaptive design facilitate the inclusion of sites from non-high-income countries, something essential in a pandemic. However, reaching confident conclusions on mortality, with an expected relatively low rate, will require large sample sizes to provide statistical power. We welcome the approach taken by the WHO in sponsoring SOLIDARITY including the participation of non-high-income countries and encourage additional non-high-income countries and sites to join the trial. The design of SOLIDARITY to allow the comparison of only the locally available medication(s) will further facilitate non-high-income countries participation. Furthermore, the recruitment of ‘several’ thousands of participants will give us confidence that it could reach relevant conclusions. RECOVERY is also a suitable trial for non-high-income countries, and we encourage the sponsor to allow this expansion. COLCOVID and ENACOVID could also be conducted in non-high-income countries. On the other hand, requesting investigators to describe the clinical status of participants on a 7- or 8-point ordinal scale or the occurrence of cases with invasive mechanical ventilation, as happened in the other seven RCTs of **Table 1**, will be difficult if not impossible in many non-high-income countries.

## EPILOGUE

Most COVID-19 RCTs are being conducted or planned to start in high-income countries and China [10]. As shown in **Table 1**, with reference to hospitalized COVID-19 patients, there are very few large non-industry RCTs assessing experimental medicines sponsored by organizations/institutions based on high-income-countries that could be suitable for non-high-income countries. To ensure the success and future value of the results, large RCTs conducted in non-high-income countries should assess generic medicines with flexible, adaptive designs. Calls for including all countries of only one region in RCTs – as the European Medicine Agency did with EU countries [11] – are needed but somewhat short-sighted in pandemics. Non-industry sponsors based in high-income countries should always bear in mind that pandemics will only be satisfactorily controlled as a global effort. Involving sites from non-high-income countries in RCTs and building their capabilities is a moral and scientific need that will pay-off in future outbreaks.

## Additional material

Online Supplementary Document
