# Supplementary material for: The need to implement non-industry COVID-19 clinical trials in non-high-income countries
Source: J Glob Health. 2020 May 15;10(1):010351. doi: 10.7189/jogh.10.010351 (PMC7242883; doi:10.7189/jogh.10.010351)
Supplement: Online Supplementary Document [file jogh-10-010351-s001.pdf]

Rafael Dal-Ré, Antonio J Carcas and Nadarajah Sreeharan **Appendix S1**

Randomized controlled trials assessing experimental medicines to treat Covid-19 hospitalized patients, that are recruiting or about to start enrollment. Trials sponsored by investigators and non-industry institutions/organizations based in high-income countries. Search on four registries (ANZCTR, ClinicalTrials.gov, EU-CTR and ISRCTN). Trials with a first registration on ANZCTR, ClinicalTrials.gov, EudraCT (accessed through EU-CTR), and ISRCTN no later than April 3, 2020.

Trial ID / Name	Country (ies) / Sponsor	Treatments[a]	N	Design / status	Primary outcome meassures
2020-001010-38 NCT04316377 / NO-COVID-19	Norway / Akershus University Hospital	Chloroquine vs SOC	202	2-arm; parallel; open-label / Not yet recruiting	Rate of decline in SARS-Covid-19-2 viral load in nasopharyngeal samples, assessed by Rt-PCR; at day 4.
2020-001113-21 ISRCTN50189673/ RECOVERY	UK / University of Oxford	Lopinavir/ritonavir vs dexamethasone vs IFNβ vs hydrochloroquine vs SOC[b]	5000	5-arm[c]; adaptive; parallel; open-label / Recruiting	In-hospital mortality; at day 28 post-randomization
2020-001224-33	Germany / Tübingen University Hospital	Hydrochloroquine vs placebo	220	2-arm; parallel; double-blind / Recruiting	Time to sustained SARS-CoC-2-specific RNA copy number ≤ 100 by Rt-PCR
2020-001254-22 / SARPAC	Belgium / Ghent University Hospital	Sargramostim vs SOC	80	2-arm; parallel; open-label / Recruiting	Sargramostim efficacy on restoring lung homeostasis by means of PaO2/FiO2 and P(A-a)O2 gradient, by day 5
2020-001602-34 NCT 04344041 / CoVitTrial	France / Angers University Hospital	Vitamin D high dose vs vitamin D standard dose	260	2-arm; parallel; open-label / Recruiting	All-cause mortality, at day 14
NCT04280705 2020-01052-18 / ACTT	Denmark, Germany, Japan, Korea, Singapore, Spain UK, USA / NIAID	Remdesivir vs placebo	572	2–arm; adaptive; parallel; double-blind; placebo-controlled / Recruiting	Percentage of subjects reporting each severity rating on an 8-point ordinal scale (from 1.death to 8.not hospitalized, no limitations on activities); by day 15
NCT04306393 2020-001490-68 2020-001329-30 / NOSARSCOVID	Austria, Sweden, USA / Massachusetts General Hospital	Nitric oxide vs no gas treatment	200	Parallel; single-blind / Recruiting	Change of arterial oxygenation (PaO2/FiO2); by day 2

NCT04305457 / NoCOVID	USA / Massachusetts General Hospital	Nitric oxide vs no gas treatment	240	Parallel; open-label / Recruiting	Reduction in the incidence of patients with mild/moderate COVID-19 requiring intubation and mechanical ventilation, by day 28
NCT04312009	USA / University of Minnesota	Losartan vs placebo	200	2-arm; parallel; double-blind / Not yet recruiting	SOFA (respiratory score), at day 28
2020-001200-42 NCT04321096 / CamoCO-19	Denmark / University of Aarhus	Camostat mesylate vs placebo	180	2-arm; parallel; double-blind / Not yet recruiting	Time to clinical improvement: days to hospital discharge or days to a 2-point improvement on a 7-point ordinal scale (from 1. Death to 7. Not hospitalized, no limitations on activities); up to discharge or death
NCT04322773 TOCIVID	Denmark / Marius Henriksen	Tocilizumab iv vs tocilizumab sc vs sarilumab sc vs SOC	200	4-arm; sequential; open-label / Not yet recruiting	Time to Independence to supplementary oxygen therapy, up to 30 days
NCT04322565	Italy / Lucio Manenti	Colchicine vs SOC	100	2-arm; parallel; open-label / Not yet recruiting	Time to clinical improvement (7-category ordinal scale), at day 28
NCT04324073 / CORIMUNO-SARI	France / Public assistance, Paris Hospitals	Sarilumab vs SOC	180	2-arm; parallel; open-label / Not yet recruiting	Survival without need of ventilator, and cumulative incidence of successful tracheal extubation, at day 14. WHO progression scale, at day 4
NCT04307693	Republic of Korea / Asan Medical Center	Lopinavir/ritonavir vs hydroxychloroquine vs SOC	150	3-arm; parallel; open-label / Recruiting	Viral load: AUC of Ct value or viral copies/mL, at days 3, 5, 7, 10, 14 and 18
2020-001156-18 / PanCovid-19	Spain / La Paz University Hospital Research Institute Foundation	Lopinavir/ritonavir vs hydroxychlo-roquine vs hydroxychloroquine + azitrhromycin	1000	3-arm; parallel; open-label / Not yet recruiting	Discharge of the patient or death (all-cause mortality)
2020–001386–37 TOCI-RE	Italy / Regio Emilia Local Health Unit–IRCCS	Tocilizumab in 3 different doses (80, 200 and 400mg)	398	2-arm; parallel; open-label / Recruiting	Occurrence of one of: 1.Need of ICU with mechanical ventilation, or 2.all-cause mortality, or 3.disease progression (PaO2/FiO2<150 mmHg)
2020-001500-41 NCT04330638 COV-AID	Belgium / Ghent University Hospital	Anakinra vs siltuximab vs anakinra + siltiximab vs tocilizumab vs anakinra + tocilizumab vs SOC	342	6-arm; factorial; open-leabel / Not yet recruiting	Time to clinical improvement (2 points on a 6-category ordinal scale or hospital discharge); at day 15
NCT04326920 SARPAC	Belgium / Ghent University Hospital	Sargramostin vs SOC	80	2-arm; parallel; open-label / Recruiting	Improvement in mean PaO2/FiO2, at day 5
2020-001301-23 NCT04325633 ENACOVID	France / Public assistance, Paris Hospitals	Naproxen vs SOC	584	2-arm; parallel; open-label / Not yet recruiting	All-cause mortality, at day 30

NCT04328480<br>COLCOVID	Argentina /ECLA	Colchicine (± lopinavir/ritonavir) vs SOC	2500	2-arm; parallel; open-label / Not yet recruiting	All-cause mortality, at day 30
NCT04328012<br>COVID MED	USA / Bassett Healthcare	Lopinavir/ritonavir vs hydrochloro-quine vs losartan vs placebo	4000	4-arm; parallel; double-blind / Not yet recruiting	NIAID Covid-19 ordinal severity scale, at day 60
NCT04325061<br>DEXA-COVID 19	Spain / Dr Negrin University Hospital	Dexamethasone vs SOC	200	2-arm; parallel; open-label / Not yet recruiting	All–cause mortality,ay day 60
NCT04327505<br>COVID-19-HBO	Sweden / Karolinska University Hospital	Hyperbaric oxygen vs SOC	200	2-arm; parallel; open-label / Not yet recruiting	PO2/FiO2 and NEWS before and after hyperbaric oxygen, and up to day 30; Clinically available markers and mechanical ventilation, up to day 30
NCT04329650	Spain / Judith Pich	Siltuximab vs methylprednisolone	100	2-arm; parallel; open-label / Not yet recruiting	Proportion of patients requiring ICU admission, at day 29
2020-001442-19<br>NCT04332094<br>TOCOVID	Spain / Santa Creu I Sant Pau Hospital Research Institute Foundation	Tocilizumab+hydroxychloroquine + azythromycin vs hydroxychloroquine+azythromycin	276	2-arm; parallel; open-label / Recruiting	In-hospital mortality, and need of mechanical ventilation; up to day 14
2020-001306-35<br>NCT04331054<br>INHASCO	France / Public assistance, Paris Hospitals	Budesonide/formoterol via inhalation vs SOC	436	2-arm; parallel; open-label / Not yet recruiting	Time to clinical improvement of two points on a 7-category ordinal scale (1. Not hospitalized with resumption of normal activities to 7. Death), up to day 30
NCT04332991<br>ORCHID	USA / Massachusetts General Hospital	Hydrochloroquine vs placebo	510	2-arm; parallel; double-blind / Not yet recruiting	Improvement on a 7-point ordinal scale (from 1. Death to 7. Not hospitalized, no limitations on activities), at day 15
NCT04332666<br>ATCO	Belgium / Erasme University Hospital	Angiotensin 1-7 vs placebo	60	2-arm; parallel; double-blind / Not yet recruiting	Composite endpoint of mortality and mechanical ventilation free days, at day 28
NCT04333368<br>STROMA-CoV2	France / Public assistance, Paris Hospitals	Umbilical cord Wharton's jelly-derived human vs NaCl 0.9%	60	2-arm; parallel; double-blind / Not yet recruiting	Increase in PaO2/FiO2 ratio from baseline, at day 7
2020-001243-15	Belgium / Leuven University Hospital	Itraconzole vs SOC	200	2-arm; parallel; open-label / Recruiting	Clinical status on a 7-point ordinal scale (from 1. not hospitalized, no limitations of activities, to 7. death); by day 15
2020-001236-10<br>COUNTER-COVID	The Netherlands / Amsterdam University Medical Center	Imatinib mesilate vs placebo	304	2-arm: parallel; single-blind / Recruiting	Composite: mortality + need for invasion + need for ECMO; at day 28
NCT04331808<br>CORIMUNO-TOCI	France / Public assistance, Paris Hospitals	Tocilizumab vs SOC	240	2-arm; parallel; open-label / Not yet recruiting	Survival without ventilator, cumulative incidence of successful tracheal extubation, at day14; WHO progression scale ≤5 and ≤7, at day 4;
2020-001246-18<br>CORIMUNO-19	France / Public assistance, Paris Hospitals	Sarilumab, tocilizumab iv vs tocilizumab sc vs SOC	1000	4-arm; parallel; open-label / Recruiting	Non-ICU patients: Survival without ventilator, at day 14, or death; and WHO progression scale < or = 5, at day 4

					ICU patients: Co-primary endpoints: 1. cumulative incidence of tracheal extubation, at day14, or death; and 2. WHO progression scale >7 at day 4
NCT04324463 ACT COVID 19	Canada / Population Health Research Institute	Azithromycin + chloroquine vs SOC	1500	2-arm; parallel; open-label / Not yet recruiting	Inpatiets: invasive mechanical ventilation or mortality; Out-patients: hospital admission or death; up to 6 weeks postrandomization
2020-001039-29 NCT04339712 ESCAPE	Greece / Hellenic Institute for the study of sepsis	Anakinra vs tocilizumab	20	2-arm; parallel; open-label / Recruiting	Composite endpoint. Achievement of at least one of: ≥25% decrease of baseline total SOFA score, or clinical improvement of lung involvement
2020-001271-33 NCT04325893 HYCOVID	France / Angers University Hospital	Hydroxychloroquine vs placebo	1300	2-arm; parallel; double-blind / Not yet recruiting	All-cause mortality or the use of intubation and invasive ventilation, at day 14
2020–000936-23 NCT04315948 / DISCOVERY	Belgium, France, Germany, Luxembourg, the Netherlands, Spain, Sweden, UK / INSERM	Remdesivir vs Lopinavir/ritonavir ± IFNβ vs vs hydroxychloroquine vs SOC	3100	5-arm[d]; adaptive; parallel; open-label / Recruiting	Clinical status on a 7-point ordinal scale (from 1. not hospitalized, no limitations of activities, to 7. death); by day 15
2020-001366-11 ISRCTN83971151 / SOLIDARITY	Argentina, Brazil, Canada, Germany, Indonesia, Iran, Norway[e], Peru, Qatar, South Africa, Spain, Switzerland, Thailand / WHO	Remdesivir vs Lopinavir/ritonavir ± IFNβ vs Hydroxy-chloroquine or chloroquine vs SOC	Thou-sands[f]	5-arm[e]; adaptive; parallel; open-label / Not yet recruiting	All-cause mortality (at discharge or death)
2020-001492-33 COVIDornase	France / Adolf de Rothschild Foundation Hospital 1 Apr	Dornase Alfa inhalation vs SOC	100	2-arm; parallel; open-label / Recruiting	Comparison of PaO2/FiO2 ratio between day 0 and day 7
2020-001445-39 NCT04341038 TRACOVID	Spain / Xavier Solanich Moreno	Methylpredinisolone + tracomilus vs SOC	84	2-arm; parallel; open-label / Recruiting	Time to clinical stability (all the following criteria are met for 48 consecutive hours: Body temperature ≤ 37.0ºC; PaO2 / FiO2> 400 and / or SatO2 / FiO2> 300; Respiratory rate ≤ 24 rpm), at day 28
2020-001457-43 COVIDICUS	France / Public assistance, Paris Hospitals	Dexamethasone + O2 vs placebo	550	8-arm; parallel; double-blind / Recruiting	In ICU patients: Overall mortality, at day 60. In non-mechanical ventilation patients: time to need of mechanical ventilation, at day 28
2020-001333-13 DHYSCO	France / Saint Joseph Paris Hospitals Group	Hydrochloroquine + dexamethasone vs hydrochloroquine	122	2-arm; parallel; open-label / Recruiting	Mortality, at day 28

2020-001278-31<br>NCT04325061<br>DEXA-COVID19	Spain / Respiratory Diseases Biomedical Research Network Center	Dexamethasone vs SOC	200	2-arm; parallel; open-label / Recruiting	All-cause mortality, at day 60
2020-001409-21<br>DEFACOVID	Spain / Foundation for Training and Health Research	Defibrotide vs placebo	120	2-arm: parallel; double-blind / Recruiting	Mortality, at day 15, 30 and 60
2020-001275-32	Denmark / The Parker Institute, Bispebjerg and Frederiksberg Hospital	Tocilizumab iv vs tocilizumab sc vs sarilumab vs SOC	200	4-arm; parallel; open-label / Recruiting	Time to Independence from O2 therapy, up to day 28
NCT04329832<br>HAHPS	USA / Intermountain Health Care	Hydroxichloroquine vs azithromycin	300	2-arm; parallel; open-label / Recruiting	Ordinal outcomes scale, at day 14
2020-001244-26<br>COV-2-SOLNATIDE-20	Austria / Medical University of Vienna	Solnatide vs placebo	20	2-arm; parallel; double-blind / Recruiting	Days free of mechanical ventilation, all-cause mortality, up to day 28. Vital signs, up to day 14. ECG & 24-fluid balance, up to day 7
2020-001406-27	France / University Hospital, Montpellier	Hydroxichloroquine + azithromycin vs hydroxichloroquine	150	2-arm; parallel; open-label / Recruiting	At least 1 level improvement on a 7-point ordinal scale (from 1. not hospitalized, no limitations of activities, to 7. death); by day 11
2020-001553-48<br>NCT04344288<br>CORTI-Covid	France / Civil Hospices, Lyon	Prednisone vs SOC	304	2-arm; parallel; open-label / Recruiting	Number of patients with SpO2<90% stabilized at rest and with O2 flowrate of ≤ 5L/min, at day 7
**Trials for specific populations**					
NCT04333407<br>C-19-ACS	UK / Imperial College London	Aspirin + clopidogrel + rivaroxabán + atrovastatin + omeprazole vs SOC	3170	2-arm; parallel; open-label / Not yet recruiting	All-cause mortality, at day 30<br><br>(trial for patients Age =/>40 or diabetes or known coronary disease or hypertension)
2020-001373-70<br>NCT04333914<br>IMMUNONCOVID	France / Leon Berard Center	Chloroquine analogue vs Nivolumab vs Tocilizumab vs SOC	273	4-arm; parallel; open-label / Not yet recruiting	Survival rate, at day 28<br>(for advanced or metastatic cancer patients)
2020-001381-11<br>NCT04329195<br>ACORES-2	France / Public assistance, Paris Hospitals	Continuation of RAS blocker vs discontinuation of RAS blocker	554	2-arm; parallel; double-blind / Not yet recruiting	Time to clinical improvement on a 7-point ordinal scale or hospital discharge; up to 28 days<br>(for patients receiving renin angiotensin blockers)

Mortality is a secondary outcome measure in all trials that do not have it as primary outcome measure, except for NCT04307693. All RCTs that were described as triple- or quadruple-blind on ClinicalTrials.gov appear in the table as double-blind to be consistent with the EU-CTR terminology, that only considers single- and double-blind trials.

AUC: Area under curve; Ct: Cycle threshold value; ECLA: Latinamerican clinical trials, Rosario; ECMO: Extracorporeal membrane oxygenation; ICU: Intensive care unit;; FiO2: Fraction of inspired oxygen; IFNβ: interferon beta; INSERM: National Institute of Health and Medical Research; NIAID: National Institute of Allergy and Infectious Diseases; PaO2: Partial pressure of oxygen in arterial blood; P(A-a)O2: Alveolar-arterial oxygen tension gradient; RAS: Renin-angiotensin-system; Rt-PCR: Reverse transcription polymerase chain reaction; SOC: standard of care; SOFA: Sequential organ failure assessment; WHO: World Health Organization.

(a)All experimental drug treatments are given on-top of standard of care; (b) Hydroxychloroquine is mentioned in the EU-CTR and in the participant's information sheet, although not in the study protocol*; (c) If one or more drugs is not available or is contraindicated, random allocation will be adjusted between the remaining arms (2:1:1 or 2:1 ratio), as stated in the protocol*; (d) It is not clear the number of arms in this trial since it has 4 on the EU-CTR (2020–000936-23; and 3300 participants) and 5 on ClinicalTrials.gov (NCT04315948; and 3100 participants); (e) It is not clear the number of arms that the trial will have in Norway. Thus, in the EU-CTR (2020-000982-18) the trial will assess only remdesivir vs hydroxychloroquine in 443 participants, but on ClinicalTrials.gov (NCT04321616), the trial will assess remdesivir vs hydroxychloroquine vs SOC in 700 participants; (f) The ISRCTN (ISRCTN83971151) registry stated 'several' thousands, whereas the EU-CTR (2020–001366-11) stated 100.000 participants (which seems a too high number).

* https://www.recoverytrial.net/professionals
